# Microglia in Alzheimer's Disease: Risk Factors and Inflammation

**DOI:** 10.3389/fneur.2018.00978

**Published:** 2018-11-15

**Authors:** Atsuko Katsumoto, Hideyuki Takeuchi, Keita Takahashi, Fumiaki Tanaka

**Affiliations:** Department of Neurology and Stroke Medicine, Yokohama City University Graduate School of Medicine, Yokohama, Japan

**Keywords:** microglia, TBI, inflammasomes, NLRP3, TREM2, neuroinflammation, glutamate

## Abstract

Microglia are resident immune cells in the central nervous system (CNS) that originate from myeloid progenitor cells in the embryonic yolk sac and are maintained independently of circulating monocytes throughout life. In the healthy state, microglia are highly dynamic and control the environment by rapidly extending and retracting their processes. When the CNS is inflamed, microglia can give rise to macrophages, but the regulatory mechanisms underlying this process have not been fully elucidated. Recent genetic studies have suggested that microglial function is compromised in Alzheimer's disease (AD), and that environmental factors such as diet and brain injury also affect microglial activation. In addition, studies of triggering receptor expressed on myeloid cells 2-deficiency in AD mice revealed heterogeneous microglial reactions at different disease stages, complicating the therapeutic strategy for AD. In this paper, we describe the relationship between genetic and environmental risk factors and the roles of microglia in AD pathogenesis, based on studies performed in human patients and animal models. We also discuss the mechanisms of inflammasomes and neurotransmitters in microglia, which accelerate the development of amyloid-β and tau pathology.

## Introduction

Alzheimer's disease (AD) is the most common neurodegenerative disease. AD brains are characterized by the combined presence of two structures: extracellular amyloid-β (Aβ) plaques and intraneuronal neurofibrillary tangles. Aβ plaques create an environment that facilitates the rapid amplification and spread of pathological tau into large aggregates, initially appearing as the neuritic, phosphorylated, microtubule-associated protein tau. This is followed by the formation and spread of neurofibrillary tangles and neuropil threads to other neurons (1).

Recent genetic studies have identified variants in immune-related genes that increase the risk of developing AD (2), implicating the neuroinflammatory response in AD pathogenesis. Notably in this regard, coding variants in the triggering receptor expressed on myeloid cells 2 (TREM2) gene confer the highest AD risk, indicating that microglial neuroinflammation plays a critical role in AD progression (3, 4). In accordance with these findings, a single-nucleotide polymorphism in the gene encoding the microglial surface receptor CD33 reduces Aβ phagocytosis by peripheral macrophages isolated from carriers of heterozygous and homozygous mutations (5–7) supporting the hypothesis that microglial function is compromised.

The microglial phenotype may change drastically over the course of neurodegeneration, as demonstrated by studies of TREM2 deficiency in a mouse model of AD (8). A recent comprehensive survey of the transcriptome of hippocampal microglia over the course of progression from the healthy to neurodegenerative state, performed at a single-cell resolution, revealed the remarkable

phenotypic heterogeneity of microglia: the early response state is characterized by marked proliferation, whereas the late response state is associated with mounting immune responses (9). In the latter state, two functionally distinct reactive microglial phenotypes, typified by modules of co-regulated type 1 and type 2 interferon response genes, have been identified (9). These functional changes in microglia are also influenced by environmental factors such as diet, brain injury, or smoking.

Here, we review how genetics and environmental factors influence microglial functions, and then illustrate the therapeutic targets in AD, with special emphasis on microglial inflammasomes and neurotransmitters.

## AD risk factors and microglia

### Genetic variants in TREM2

TREM2 is a type I transmembrane receptor expressed in a subset of myeloid lineage cells including microglia, dendritic cells, osteoclasts, monocytes, and tissue macrophages (10, 11). Homozygous mutations in *TREM2* cause Nasu-Hakola disease, and rare heterozygous variants are associated with other neurodegenerative diseases such as late-onset AD (3, 4), frontotemporal dementia (12), and Parkinson's disease (13). Although the exact molecular mechanisms underlying the development of neurodegeneration in the brain remain unknown, abnormalities in TREM2 and its interacting partner DNAX activating protein of 12 kDa (DAP12) appear to cause dysregulation of microglial inflammatory responses and neuronal debris clearance (14, 15). In addition, TREM2 affects microglial survival in an AD mouse model, as TREM2-deficient microglia are not able to sustain microgliosis and undergo apoptosis rather than becoming activated (15). Transcriptome analysis also revealed the role of TREM2 in chemotaxis, migration, and mobility (16), as TREM2 deficiency results in ineffective plaque encapsulation of Aβ and reduced plaque compaction, which is associated with worsened axonal pathology. Data from TREM2 knockout mice revealed that CCL2, IL-1β, TNF-α, and secreted phosphoprotein 1 (SPP1) are the direct targets of TREM2 signaling (16). Furthermore, TREM2 deficiency influences the microglial metabolic state through the mammalian target of rapamycin pathway (17). Microglia lacking TREM2 undergo global changes in their metabolism, resulting in reduced ATP levels and signs of stress and death. These observations imply that TREM2 is a critical regulator of microglial phenotypes.

Interestingly, TREM2 plays distinct functional roles at different stages: in a mouse model of AD, TREM2 deficiency ameliorates amyloid pathology in the early disease stage, but exacerbates the pathology as the disease progresses (8). One possible explanation might be that TREM2 deficiency affects different myeloid cell subsets at different stages of AD pathology. TREM2 deficiency first affects CD45^hi^ myeloid cells, where it is primarily expressed, but subsequent loss of these CD45^hi^ cells also affects the function of CD45^lo^ myeloid cells, decreasing their proliferation and potentially altering other AD-related phenotypes. Regarding the change in microglial phenotypes, immune memory in microglia has been shown to modify Aβ pathology in AD mice (18), in which repeated stimulation shift from inflammatory to phagocytic microglia by differential epigenetic reprogramming. The blocking of epigenetic factors enhanced immune training in microglia, decreases Aβ levels and improves memory in AD mice (19).

**Figure 1:**
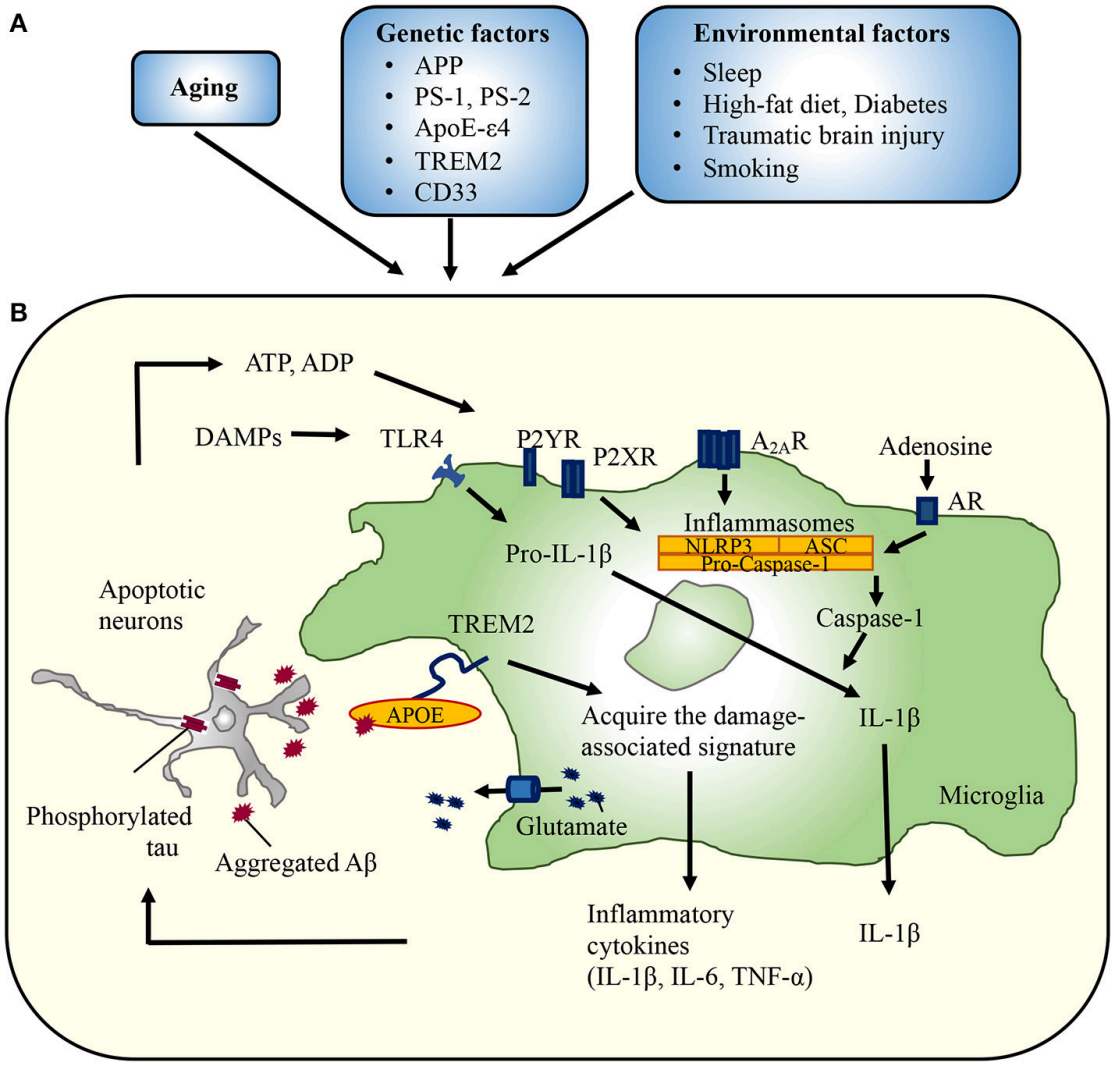
Implication of microglia in the development of Alzheimer's disease. **(A)** Several conditions are associated with an increased risk of developing AD. For example, variants in TREM2 ameliorate amyloid pathology in the early disease stage, but exacerbate pathology as the disease progresses. The TREM2-APOE pathway is responsible for switching from a homeostatic to a neurodegenerative microglial phenotype after phagocytosis of apoptotic neurons. Environmental factors also affect the microglial reaction to aggregated Aβ or phosphorylated tau. Head trauma also leads to a local increase in the levels of inflammatory mediators, which may stimulate Aβ generation and restrict phagocytic clearance. Likewise, microbiota influenced by diabetes or diet may regulate microglial phenotypes. **(B)** Aggregated Aβ or phosphorylated tau impairs synaptic functions, triggering the release of neurotoxic mediators from microglia. ATP, ADP, and adenosine activate NLRP3 inflammasomes, followed by the release of IL-1β. Similarly, glutamate released from gap junction hemichannels lead to massive neuronal damage. APP, amyloid precursor protein; PS, presenilin; ApoEε4, apolipoprotein Eε4; TREM2, triggering receptor expressed on myeloid cells 2; P2X(Y)R, purinergic receptor; A_2A_Rs, adenosine A_2A_ receptors; AR, adenosine receptor; DAMPs, damage-associated molecular patterns; NLRP3, NACHT, LRR, and PYD domains-containing protein 3; ASC, apoptosis-associated speck-like protein containing a caspase-recruitment domain.

Recent studies reported that binding of apolipoproteins including apolipoprotein E (APOE) with TREM2 facilitates microglial uptake of Aβ (20) and that the TREM2-APOE pathway was identified as the mechanism responsible for switching from a homeostatic to a neurodegenerative microglial phenotype after phagocytosis of apoptotic neurons (21) (Figure 1). Targeting the TREM2-APOE pathway restored the homeostatic signature of microglia in AD mouse models and prevented neuronal loss in an acute model of neurodegeneration (21). Moreover, the APOE-mediated neurodegenerative microglia lost their tolerogenic function. These findings imply that the TREM2-APOE pathway is a major regulator of the microglial functional phenotype in neurodegenerative diseases. On the contrary, the transition from homeostatic microglia expressing *Cx3cr1, P2ry12*, and *Tmem119* to the disease-associated microglia (DAM) state with induction of *ApoE* was independent of TREM2 (22). Following loss of homeostatic signature, microglia increase phagocytic and lipid metabolism activity including upregulation of *TREM2* and *lipoprotein lipase* to be the full DAM, which depends on TREM2. Moreover, loss of microglial CX3CR1 has opposing effects Aβ and tau pathologies (23, 24). Further studies are needed to uncover the precise mechanism of TREM2-APOE pathway in AD pathology.

These studies were performed on animal models of Aβ-related pathologies, but little is known regarding the role of TREM2 in regulating intracellular tau pathology. Elevated levels of soluble TREM2 in the cerebrospinal fluid (CSF) of AD patients, as determined by mass spectrometry, are correlated with levels of CSF total tau and phosphorylated-tau, but not the level of CSF Aβ42 (25). Notably in this regard, CSF analysis revealed that a recently reported rare variant in TREM2 (p.R47H, rs75932628) is significantly associated with the risk of AD (26). In addition, carriers of the risk allele exhibited similar phenotypes (significantly elevated levels of CSF total tau, but not Aβ42, in AD patients). In addition, our group has recently reported that TREM2 deficiency leads to heightened tau pathology coupled with widespread activation of neuronal stress kinases, including ERK1/2 and JNK, in a mouse model of tauopathy (27). These observations support the hypothesis that CSF TREM2 is a marker for tau dysfunction in AD.

### Traumatic brain injury (TBI)

TBI is associated with the development of neurodegenerative conditions such as AD and chronic traumatic encephalopathy. A prominent feature of TBI is the development of an inflammatory reaction within minutes of the injury event. Damage-associated molecular patterns (DAMPs) (e.g., ATP, reactive oxygen species, damaged mitochondria, and necrotic cells) activate microglia and resident mononuclear phagocytes in the CNS, which promote neuroprotection and repair through the clearance of tissue debris and subsequent resolution of the inflammatory response (28, 29). Unless properly controlled, microglial activity leads to further neuronal damage through secretion of pro-inflammatory cytokines and reactive species, as well as, other mechanisms (29). Analysis of mRNA expression in microglia/macrophages revealed a rapid rise and fall in the protective phenotype (CD206, Arg1, Ym1/2, and TGF-β) and a sustained rise in the inflammatory phenotype (iNOS, CD11b, CD16, and CD86) after TBI (30). On the other hand, blocking neural/microglial interaction via CX3CR1 deficiency conferred neurological protection at early time points after TBI, but caused appreciable impairments accompanied by persistent neuronal death at later times (31, 32). *In vivo* imaging with positron emission tomography for activated microglia in patients revealed elevated microglial activation for several years after TBI (33).

How, then, can microglia activated by TBI trigger rapid and insidiously progressive AD-like pathological changes? Elevation of the Aβ burden and phosphorylated tau has been observed in patients within hours after TBI (34, 35). TBI-induced axonal injury is among the first perturbations of tau that results in dissociation from microtubules. *Cis* phosphorylated-tau (p-tau) appears within hours after closed head injury and long before other known pathogenic p-tau conformations, including oligomers, pre-fibrillary tangles, and NFTs (36). In particular, *cis* p-tau contributes to functional impairment in an animal model of TBI, as well as, in humans (37). Murine microglia rapidly internalize and degrade hyperphosphorylated tau (38), and expression of tau by microglia themselves also promotes their activation (39). Thus, robust and persistent inflammation may be sufficient to promote tauopathy.

Microglia may play a dual role in Aβ accumulation and clearance. Increased expression of the gamma secretase complex proteins on microglia and astrocytes have been observed in a closed head injury model (40). On the other hand, microglia containing Aβ have been found in association with plaques after TBI (41), suggesting phagocytic clearance of Aβ by proteases such as neprilysin and insulin-degrading enzyme (42). Suppression of microglial activation is associated with decreases in TBI-induced Aβ and restores depressed neurogenesis (43). It should be noted, however, that no studies have conclusively determined whether Aβ is the cause of microglial activation and inflammation following TBI.

Given that recent clinicopathologic and biomarker studies have failed to confirm the relationship between TBI and development of AD dementia or pathologic changes (44–46), it is possible that TBI exposure is a risk for late-life neurodegeneration but not AD. Therefore, further investigation is clearly needed to determine the relationship between TBI and cognitive decline.

### Gut-brain axis

Recent studies have revealed the relationship between the gastrointestinal tract and the brain. Germ-free mice exhibit global defects in microglia with altered cell proportions and an immature phenotype, leading to impaired innate immune responses. Limited microbiota complexity also resulted in dramatic alterations in microglial properties (47). In addition, short-chain fatty acids and microbiota-derived bacterial fermentation products, have been demonstrated to regulate microglia maturation and function (47). In AD mice, perturbations in microbial diversity following antibiotic exposure diminish amyloid pathology (48). Microglia, which lie at the interface between environmental signals and brain circuitry throughout embryonic and adult life, are prime candidates as mediators of these effects.

### Sleep

Lack of sleep is suggested as a risk for AD. Chronic lack of sleep increases Aβ plaque deposition (49), and sleep promotes efficient soluble Aβ clearance (50). Lack of sleep affects microglial morphology, phagocytosis, and Aβ clearance (51, 52). A recent study revealed that upregulation of complement C1q and C3 promotes synapse loss by microglial phagocytosis in AD (53). Even a short period of sleep loss enhances the mouse cerebral cortex expression level of complement C3 which activates synapse loss by microglia, and impaired sleep-wake cycle reduces microglial Aβ clearance (51). Moreover, chronic sleep restriction, but not acute sleep deprivation, promotes microglial phagocytosis without neuroinflammation (52). More detailed studies are needed to clarify how sleep affects microglial function and AD pathogenesis.

## Inflammatory cues in AD

### Inflammasomes

Inflammasomes are a group of cytosolic protein complexes that form to mediate host immune responses to microbial infection and cellular damage (54). Assembly of an inflammasome triggers proteolytic cleavage of dormant procaspase-1 into active caspase-1, which converts IL-1 family cytokine precursors, pro-IL-1β, and pro-IL-18, into mature and biologically active IL-1β and IL-18, respectively (55). IL-1β and IL-18, in turn, initiate multiple signaling pathways and drive inflammatory responses, which results in neuronal injury or death (Figure 1).

Because IL-1β and IL-18 are key contributors to the progression of chronic inflammation—associated neurodegenerative diseases, including AD, inflammasomes are considered to be major players in chronic neuroinflammation (56, 57). The Aβ oligomer promotes the processing of pro-IL-1β into mature IL-1β in microglia, which in turn enhances microglial neurotoxicity (57). Levels of nucleotide-binding oligomerization domain-, leucine-rich repeat-, and pyrin domain-containing 3 (NLRP3) inflammasomes and caspase-1 are substantially elevated in the brains of AD patients (56, 58), and elevated expression of IL-1β and IL-18 initiates inflammatory processes in the brain of AD patients. Elevated expression of these cytokines has also been detected in microglia and astrocytes, as well as, in neurons, co-localized with both Aβ plaques and tau deposition. Chronic inflammation may be responsible for increases in Aβ accumulation and tau phosphorylation in the brain (59). Halle et al. identified the NLRP3 inflammasome as a sensor of Aβ in a process involving phagocytosis of Aβ and subsequent lysosomal damage and release of cathepsin B (60).

Damaged neurons injured by insoluble Aβ oligomers and fibrils release DAMPs, which are sensed by NLRP3 inflammasomes, initiating a chain of events that leads to the maturation of pro-IL-1β and pro-IL-18 and release of their active forms (Figure 1) (60, 61). In addition, NLRP3 inflammasomes sense disease-associated extracellular amyloid and unique protein aggregates caused by inappropriate oligomerization or misfolding (62), likely as DAMPs within the resident microglia/macrophages after engulfment in the brain. Deficiency of NLRP3 or caspase-1 substantially attenuates spatial memory impairment and enhances Aβ clearance in AD model mice, indicating the importance of inflammasome-mediated neuroinflammation in AD pathogenesis (56). Furthermore, upon activation, microglia release ASC specks (63). These bodies have a direct molecular link to classical hallmarks of neurodegeneration: ASC specks bind to Aβ in the extracellular space and promote its aggregation, thereby directly activating innate immunity in association with the progression of AD pathology. Lysates derived from APP/PS1;Asc^−/−^ brains had a reduced capacity to increase the Aβ load. Furthermore, a specific anti-ASC antibody prevented Aβ aggregation (63). Given that tau oligomers are known to spread to neighboring cells, their relationship to inflammasome activation should be examined further. Of interest, recent data emphasize that pathological tau promotes IL-1β secretion by activating inflammasomes.

### Neurotransmitters

Microglia are closely associated with astrocytes and neurons, particularly at synapses, and recent data indicate that neurotransmitters play an important role in regulating the morphology and function of surveying/resting microglia, which express receptors for most known neurotransmitters (64, 65). In particular, microglia express receptors for ATP and glutamate, which regulate their motility. When Aβ induces ATP secretion by neurons (66) and microglia (67), effector functions such as phagocytosis and cytokine secretion are triggered.

Glutamate clearance and regulation at synaptic clefts is primarily mediated by glial transporter 1, and that expression is reduced in human AD hippocampal tissue (68). Consequently, glutamate overload triggers synaptic and neuronal loss influenced by AMPA receptors, which potentially contributes to AD. Levels of AMPA receptor subunit GluA2 are reduced in accordance with the Braak stages of AD (69). Lack of GluA2 in microglia leads to Ca^2+^ permeability in response to glutamate and may cause excess release of inflammatory cytokines, thereby increasing glutamate toxicity to neurons. Inhibition of glutamate receptor signaling has been proposed as a therapeutic approach for several neurodegenerative diseases. Because gap junctions/hemichannels are the main avenues for release of excessive glutamate from neurotoxin-activated microglia (70), their blockade by glycyrrhetinic acid derivatives significantly prevents activated microglia/macrophage-mediated neuronal death in rodent models of AD (71, 72). Moreover, because gap junctions/hemichannels are the main source of ATP, UTP, and glutamate, their blockade can halt the vicious cycle of transmission and amplification of neuroinflammation, and this also represents a promising therapeutic strategy for CNS diseases (65).

Adenosine A_2A_ receptors (A_2A_Rs) expressed by astrocytes and microglia are at the center of a neuromodulatory network that interacts with and integrates several neurotransmitter pathways. A_2A_Rs modulate both glial activation and the ability of glia to release inflammatory factors or take up glutamate (73), and also mediates microglial process retraction (74). Expression of A_2A_R in microglial cells is elevated in the hippocampus and cerebral cortex of AD patients (75). Interestingly, consumption of caffeine, a non-selective adenosine A_2A_Rs antagonist, reduces the risk of developing AD (76) and mitigates both amyloid and tau burden in transgenic mouse models (77, 78). Blockade of adenosine A_2A_Rs decreases both hippocampal tau phosphorylation and neuroinflammatory response in a tauopathy mouse model (79), and also decreases amyloid burden in the brain and improves cognitive performance in an Aβ-injection model (80, 81). Therefore, regulation of inflammatory responses by microglial transmitters may have effects on AD.

## Conclusions

Here, we briefly discussed the role of microglial functions in the development of AD. Microglial reactions in neurological disorders are complex and vary among disease stages; indeed, pro-inflammatory and anti-inflammatory microglia co-exist in some contexts. Newly emerging data reveal that microglia are a unique cell-population, to which the simple M1/M2 classification does not fit. Further investigation focusing on the microglial regulation will be required to develop new therapeutic interventions targeting CNS neuroinflammatory pathways.

## Author contributions

AK wrote the manuscript. HT, KT, and FT edited the manuscript.

### Conflict of interest statement

The authors declare that the research was conducted in the absence of any commercial or financial relationships that could be construed as a potential conflict of interest.

## Abbreviations

A_2A_Rs: adenosine A_2A_ receptors
ASC: apoptosis-associated speck–like protein containing a caspase-recruitment domain
DAMPs: damage-associated molecular patterns
NLRP3: nucleotide-binding oligomerization domain-, leucine-rich repeat–and pyrin domain–containing 3
TBI: traumatic brain injury
TREM2: triggering receptor expressed on myeloid cells 2.
